# Lessening Organ Dysfunction With Vitamin C (LOVIT) Trial: Statistical Analysis Plan

**DOI:** 10.2196/36261

**Published:** 2022-05-20

**Authors:** Neill KJ Adhikari, Ruxandra Pinto, Andrew G Day, Marie-Hélène Masse, Julie Ménard, Sheila Sprague, Djillali Annane, Yaseen M Arabi, Marie-Claude Battista, Dian Cohen, Deborah J Cook, Gordon H Guyatt, Daren K Heyland, Salmaan Kanji, Shay P McGuinness, Rachael L Parke, Bharath Kumar Tirupakuzhi Vijayaraghavan, Emmanuel Charbonney, Michaël Chassé, Lorenzo Del Sorbo, Demetrios James Kutsogiannis, François Lauzier, Rémi Leblanc, David M Maslove, Sangeeta Mehta, Armand Mekontso Dessap, Tina S Mele, Bram Rochwerg, Oleksa G Rewa, Jason Shahin, Pawel Twardowski, Paul Jeffrey Young, François Lamontagne, LOVIT Investigators

**Affiliations:** 1 Department of Critical Care Medicine Sunnybrook Health Sciences Centre Toronto, ON Canada; 2 Interdepartmental Division of Critical Care Medicine University of Toronto Toronto, ON Canada; 3 Kingston Health Sciences Centre Kingston, ON Canada; 4 Department of Public Health Sciences Queen's University Kingston, ON Canada; 5 Research Centre of the Centre Hospitalier Universitaire de Sherbrooke Sherbrooke, QC Canada; 6 Division of Orthopaedic Surgery Department of Surgery McMaster University Hamilton, ON Canada; 7 Department of Intensive Care Medicine Raymond-Poincaré Hospital (Assistance Publique - Hôpitaux de Paris [AP-HP]) Garches France; 8 Institut National de la Santé et de la Recherche Médicale Université Paris-Saclay Campus Université de Versailles Saint-Quentin-en-Yvelines Versailles France; 9 Fédération Hospitalo-Universitaire SEPSIS Garches France; 10 College of Medicine King Saud Bin Abdulaziz University for Health Sciences Riyadh Saudi Arabia; 11 King Abdullah International Medical Research Center Riyadh Saudi Arabia; 12 Department of Intensive Care King Abdulaziz Medical City Ministry of National Guard Health Affairs Riyadh Saudi Arabia; 13 Department of Medicine Université de Sherbrooke Sherbrooke, QC Canada; 14 Bishop's University Sherbroooke, QC Canada; 15 Massawippi Valley Foundation Ayer's Cliff, QC Canada; 16 Department of Medicine McMaster University Hamilton, ON Canada; 17 Department of Critical Care St. Joseph's Healthcare Hamilton Hamilton, ON Canada; 18 Department of Health Research Methods, Evidence, and Impact McMaster University Hamilton, ON Canada; 19 Department of Critical Care Medicine Queen's University Kingston, ON Canada; 20 Kingston General Health Research Institute Kingston Health Sciences Centre Kingston, ON Canada; 21 Clinical Evaluation Research Unit Kingston General Hospital Kingston, ON Canada; 22 The Ottawa Hospital Research Institute Ottawa, ON Canada; 23 Department of Pharmacy The Ottawa Hospital Ottawa, ON Canada; 24 Cardiothoracic and Vascular Intensive Care Unit Auckland City Hospital Auckland New Zealand; 25 Medical Research Institute of New Zealand Newtown New Zealand; 26 Australian and New Zealand Intensive Care Research Centre Monash University Melbourne Australia; 27 School of Nursing Faculty of Medical and Health Sciences University of Auckland Auckland New Zealand; 28 Department of Critical Care Medicine Apollo Hospitals Chennai India; 29 The George Institute for Global Health New Delhi India; 30 Centre Hospitalier de l'Université de Montréal Montréal, QC Canada; 31 Centre de Recherche de l'Hôpital du Sacré-Coeur de Montréal Montréal, QC Canada; 32 Department of Medicine Université de Montréal Montréal, QC Canada; 33 Department of Medicine University Health Network Toronto, ON Canada; 34 Department of Critical Care Medicine University of Alberta Edmonton, AB Canada; 35 Department of Medicine Université Laval Québec, QC Canada; 36 Department of Anesthesiology and Critical Care Université Laval Québec, QC Canada; 37 Population Health and Optimal Health Practices Research Unit, Traumatology - Emergency - Critical Care Medicine Centre Hospitalier Universitaire de Québec - Université Laval Research Center Centre Hospitalier Universitaire de Québec - Université Laval Québec, QC Canada; 38 Critical Care Medicine Service Centre Hospitalier Universitaire de Québec - Université Laval Quebec, QC Canada; 39 Department of Medicine Centre Hospitalier Universitaire Dr Georges-L. Dumont Moncton, NB Canada; 40 Division of Intensive Care Centre Hospitalier Universitaire Dr Georges-L. Dumont Moncton, NB Canada; 41 Department of Medicine Queen's University Kingston, ON Canada; 42 Sinai Health System Toronto, ON Canada; 43 Service de Médecine Intensive Réanimation Assistance Publique - Hôpitaux de Paris [AP-HP], Hôpitaux Universitaires Henri-Mondor Créteil France; 44 Groupe de Recherche Clinique CARMAS (Cardiovascular and Respiratory Manifestations of Acute lung injury and Sepsis) Université de Paris Est Créteil Créteil France; 45 Institut Mondor de Recherche Biomédicale Institut National de la Santé et de la Recherche Médicale Université Paris Est Créteil Créteil France; 46 Department of Surgery Schulich School of Medicine and Dentistry Western University London, ON Canada; 47 Department of Medicine McGill University Health Centre Montréal, QC Canada; 48 Department of Critical Care McGill University Health Centre Montréal, QC Canada; 49 Department of Surgical Sciences University of Otago Dunedin New Zealand; 50 Intensive Care Unit Wellington Hospital Newton New Zealand; 51 See Acknowledgements

**Keywords:** sepsis, vitamin C, statistical analysis, organ, ascorbic acid, critical care, organ dysfunction, intensive care unit, intensive care, patient, vasopressor, infection, intravenous, health data, trial database, patient outcome, mortality, statistical framework, binomial distribution

## Abstract

**Background:**

The LOVIT (Lessening Organ Dysfunction with Vitamin C) trial is a blinded multicenter randomized clinical trial comparing high-dose intravenous vitamin C to placebo in patients admitted to the intensive care unit with proven or suspected infection as the main diagnosis and receiving a vasopressor.

**Objective:**

We aim to describe a prespecified statistical analysis plan (SAP) for the LOVIT trial prior to unblinding and locking of the trial database.

**Methods:**

The SAP was designed by the LOVIT principal investigators and statisticians, and approved by the steering committee and coinvestigators. The SAP defines the primary and secondary outcomes, and describes the planned primary, secondary, and subgroup analyses.

**Results:**

The SAP includes a draft participant flow diagram, tables, and planned figures. The primary outcome is a composite of mortality and persistent organ dysfunction (receipt of mechanical ventilation, vasopressors, or new renal replacement therapy) at 28 days, where day 1 is the day of randomization. All analyses will use a frequentist statistical framework. The analysis of the primary outcome will estimate the risk ratio and 95% CI in a generalized linear mixed model with binomial distribution and log link, with site as a random effect. We will perform a secondary analysis adjusting for prespecified baseline clinical variables. Subgroup analyses will include age, sex, frailty, severity of illness, Sepsis-3 definition of septic shock, baseline ascorbic acid level, and COVID-19 status.

**Conclusions:**

We have developed an SAP for the LOVIT trial and will adhere to it in the analysis phase.

**International Registered Report Identifier (IRRID):**

DERR1-10.2196/36261

## Introduction

Sepsis, defined as a dysregulated host immune response to infection that leads to organ dysfunction and death [1], is a major global public health concern, causing up to 5.3 million deaths every year. Current sepsis management is focused on prompt antimicrobial therapy and organ-supportive care, and numerous trials of interventions for immune dysregulation have not demonstrated benefit [2]. Vitamin C is an endogenous antioxidant with multiple actions, including scavenging of oxygen radicals, restoration of endothelial function, and synthesis of norepinephrine and vasopressin as a cofactor. The findings of low vitamin C levels in critical illness and its association with poor outcomes have led to randomized clinical trials (RCTs) of intravenous vitamin C [3], including in sepsis [4], with variable results that do not exclude clinically meaningful improvements in patient outcomes.

The LOVIT (Lessening Organ Dysfunction with Vitamin C) trial is the largest trial to evaluate high-dose intravenous vitamin C in adults with sepsis. We aim to describe a prespecified statistical analysis plan (SAP) for the LOVIT trial. This SAP was written before data collection was complete for the last adult enrolled in the trial and prior to database lock and unblinding of the study team.

## Methods

### Design

The LOVIT trial is a multicenter, parallel-group, allocation-concealed, blinded (participants, clinicians, study personnel, members of the executive and steering committees, and data analysts) superiority RCT, which was registered on September 21, 2018 (ClinicalTrials.gov identifier: NCT03680274). The trial protocol has been published [5]; the final version (7.0) is dated February 15, 2021. The primary aim of the LOVIT trial is to determine whether intravenous vitamin C, administered to adults with sepsis receiving a vasopressor, reduces the composite outcome of mortality and persistent organ dysfunction [6] at day 28, when compared with placebo. Persistent organ dysfunction is defined as dependency on vasopressors, mechanical ventilation, or incident renal replacement therapy.

### Sites

The trial involves 35 sites in Canada, New Zealand, and France.

### Inclusion Criteria

Patients were eligible for inclusion if they were (1) at least 18 years old; (2) admitted to an intensive care unit (ICU) with proven or suspected infection as the main diagnosis; and (3) treated with a continuous intravenous vasopressor infusion (norepinephrine, epinephrine, vasopressin, dopamine, or phenylephrine [or metaraminol in New Zealand]) at the time of eligibility assessment and at randomization.

The LOVIT trial was designed before the COVID-19 pandemic, but patients with SARS-CoV-2 infection who otherwise met the eligibility criteria were eligible for the trial.

### Exclusion Criteria

Patients were excluded for any of the following reasons: (1) more than 24 hours since ICU admission; (2) known glucose-6-phosphate dehydrogenase deficiency; (3) pregnancy; (4) known allergy to vitamin C; (5) known kidney stones within the past 1 year; (6) received any intravenous vitamin C during the current hospitalization, unless incorporated as part of parenteral nutrition; (7) expected death or withdrawal of life-sustaining treatments within 48 hours; (8) previously enrolled in this study (LOVIT trial); and (9) enrolled in a trial for which co-enrollment was not possible (determined on a case-by-case basis by discussion with the other trial’s principal investigators).

### Randomization

Trial participants were randomized in a 1:1 ratio to vitamin C or matching placebo using permuted blocks of variable size, undisclosed to study personnel, and stratified by clinical site using a web-based randomization interface. Pharmacists and technicians preparing the study medication (vitamin C or placebo) at each participating site were unblinded.

### Intervention

The experimental intervention was intravenous vitamin C, administered in bolus doses of 50 mg/kg actual body weight, given every 6 hours for 96 hours (ie, 200 mg/kg/day and 16 doses in total), as long as the patient remained in the ICU. For patients weighing ≥150 kg, the weight was considered as 150 kg to calculate the dose. Each dose was administered over 30-60 minutes, except for participants >120 kg, for whom the infusion time was prolonged so that the rate did not exceed 100 mg/min. Participants in the control arm received 5% dextrose or normal saline in a volume to match the vitamin C. Placebo was infused over the same period as per the instructions for vitamin C, and was identical in color and other physical properties to vitamin C. Administration of open-label vitamin C in either group was not permitted and constituted a protocol violation.

### Primary Outcome

The primary outcome is a composite of mortality and persistent organ dysfunction (defined as dependency on vasopressors, mechanical ventilation, or new renal replacement therapy) at day 28 [6]. Mechanical ventilation refers to invasive ventilation only, and patients receiving chronic renal replacement therapy before the index hospitalization do not meet the criteria for persistent organ dysfunction on the basis of ongoing renal replacement therapy. Note that day 1 refers to the day of randomization.

### Secondary Outcomes

Efficacy outcomes include the following:

1. Persistent organ dysfunction–free days in the ICU, defined as the number of days alive and not dependent on vasopressors, mechanical ventilation, or new renal replacement therapy, up to day 28 and while in the ICU. Patients who die on or before day 28 will be assigned a value of −1 (modified from a previous report [7]). Any patient not receiving renal replacement therapy on a given day will be counted as renal replacement therapy–free for that day, even if renal replacement therapy is delivered on the day before or after this renal replacement therapy–free day. For patients on chronic renal replacement therapy before ICU admission, renal replacement after randomization will not be counted as organ dysfunction. Patients discharged from the ICU to a hospital ward before day 28 and who receive renal replacement therapy after ICU discharge will not be counted as having persistent organ dysfunction after ICU discharge. Patients discharged from the study ICU to another hospital’s ward or ICU before day 28 and not receiving these interventions at discharge will be assumed to not be receiving them at day 28 if specific information is unavailable. Similarly, patients discharged from the study ICU to another hospital’s ICU before day 28 and receiving any of these interventions at discharge from the study ICU will be assumed to be receiving them at day 28 if specific information is unavailable.
2. Mortality at 6 months.
3. Health-related quality of life (HRQoL) in 6-month survivors, as assessed using the 5-level EuroQol 5 dimensions [8] questionnaire. This scale evaluates mobility, personal care, usual activities, pain/discomfort, and anxiety/depression, and categorizes each of these dimensions into 5 levels that range from no problems to extreme problems. Respondents also evaluate their overall health status using a 100-point scale.
4. Global tissue dysoxia assessed at days 1, 3, and 7, measured by serum lactate levels [9]. This is assessed using liquid chromatography coupled with tandem mass spectrometry.
5. Organ function (including renal function) assessed by the sequential organ failure assessment (SOFA) score [10] at days 1, 2, 3, 4, 7, 10, 14, and 28. The SOFA score on day 1 may have included physiological data obtained after administration of study medication.
6. Inflammation at days 1, 3, and 7, assessed by serum interleukin-1 beta, tumor necrosis factor-alpha, and C-reactive protein levels, measured by Luminex (Luminex Corp).
7. Infection biomarker (serum procalcitonin [11]) levels at days 1, 3, and 7, measured using an enzyme-linked immunosorbent assay.
8. Endothelial injury at days 1, 3, and 7, assessed by serum thrombomodulin [11] and angiopoietin-2 levels [12], measured by Luminex.

Biomarker outcomes were measured only in patients enrolled in Canada. We included day 1 measurements of biomarkers in the outcome list above for completeness, although day 1 samples were taken before administration of the first dose of study medication, and these samples therefore provided baseline measurements. Biomarker analyses were conducted in a central study laboratory; due to delays in obtaining assays to measure procalcitonin and C-reactive protein, analyses of those secondary outcomes may be delayed and reported after the primary publication.

Safety outcomes include the following:

1. Stage 3 acute kidney injury as defined by Kidney Disease-Improving Global Outcomes criteria [13], using either serum creatinine or urine output criteria, at any time during the ICU stay.
2. Acute hemolysis, ascertained until 12 hours after the last dose of study medication, defined as clinician judgment of hemolysis, as recorded in the chart, or a hemoglobin drop of at least 25 g/L within 24 hours of a dose of study medication and 2 of the following: reticulocyte count >2 times the upper limit of normal; haptoglobin less than the lower limit of normal; indirect (unconjugated) bilirubin >2 times the upper limit of normal; or lactate dehydrogenase >2 times the upper limit of normal. Normal values are as defined at each participating center’s laboratory. Severe hemolysis is defined as hemoglobin <75 g/L, at least 2 of the above criteria, and the requirement for transfusion of at least 2 units of packed red blood cells. As a secondary assessment of this acute hemolysis and of severe hemolysis, medical records of patients flagged as having hemolysis will be adjudicated by 2 blinded steering committee members, and any patient with hemolysis judged at least possibly related to the study drug after adjudication will be counted.
3. Hypoglycemia, defined as a blood glucose level measured in the hospital core laboratory of less than 3.8 mmol/L. Vitamin C therapy may be associated with falsely elevated glycemic readings when certain point-of-care glucometers are used to measure blood glucose [14]. Because elevated glycemic values may prompt iatrogenic hypoglycemic episodes if insulin or oral hypoglycemic agents are administered, hypoglycemic events will be reported as a safety outcome.

After trial registration and publication of the trial protocol [5], we added the secondary outcome of mortality at 28 days, which is a component of the primary outcome. The trial registration reports 3 other outcomes (vitamin C volume of distribution, clearance, and plasma concentration) that are only relevant for a pharmacokinetic substudy, whose analysis plan will be reported separately.

### Adverse Events

Following Canadian recommendations for adverse event reporting in academic critical care trials [15], expected adverse events (death, stage 3 acute kidney injury, hemolysis, and hypoglycemia), whether severe or not, are prespecified trial outcomes and will not be reported separately as adverse events. Unexpected adverse events that are serious (ie, fatal, life-threatening, prolonging hospital stay, resulting in persistent or significant disability or incapacity, or constituting an important medical event according to the local principal investigator) and considered by the local principal investigator to be at least possibly related to trial procedures will be reported to the coordinating center within 24 hours of becoming aware of the event.

### Sample Size

We determined a minimum sample size of 800 participants based on the following assumptions. We established that an absolute difference of 10% in the composite outcome of mortality and persistent organ dysfunction (15% to 25% relative risk reduction) would be plausible [16,17] and sufficiently large to change practice. Based on recent clinical trials in a similar population [18], the risk of 28-day persistent organ dysfunction or mortality in the control arm was expected to be approximately 50%. By enrolling 385 evaluable patients per arm, the study would have 80% power to detect a 10% absolute risk reduction (from 50% to 40%, which corresponds to a 20% relative risk reduction). To account for consent withdrawal and loss to follow-up, we planned to enroll 400 patients per arm. Because of the subsequent COVID-19 pandemic, which started after the LOVIT trial had commenced recruiting and constituted extenuating circumstances [19], the steering committee approved the inclusion of eligible patients in whom SARS-CoV-2 infection was the cause of sepsis. However, in view of the unclear responsiveness of sepsis in the context of COVID-19 to vitamin C, the total sample size was increased to ensure that the original planned sample size (n=800) of non–COVID-19 participants was reached. We have planned a subgroup analysis based on COVID-19 status (mentioned below).

### Statistical Analysis

#### Interim Analyses

The independent Data and Safety Monitoring Committee (DSMC) reviewed data on all serious unexpected adverse events at least possibly related to the study medication, in addition to hemolysis, stage 3 acute kidney injury, and hypoglycemia, after enrollment of 250 and 530 patients. The statistical plan for the interim analyses was included in the DSMC charter, which was written before enrollment of the first patient in the trial, and included in the protocol [5]. In an unadjusted analysis using a chi-square or Fisher exact test as appropriate, if the 1-sided *P* value had been <.1 (in the direction of harm in the vitamin C arm) for any of the 3 safety outcomes, an interim 2-sided analysis of the primary outcome would have been conducted. The DSMC could also have requested an analysis of the primary outcome at any time. This analysis would have generated a conditional power for showing statistically significant efficacy (superiority of vitamin C) in the final analysis of the primary outcome, assuming that the group-specific event rates observed to date had remained the same in the total sample size. If the conditional power for efficacy had been <20%, in the context of a 1-sided *P* value <.1 for any of the safety outcomes, the DSMC could have recommended stopping the trial to the steering committee. The DSMC could have made a similar recommendation even if these exact thresholds had not been met, based on its interpretation of the balance between safety and efficacy. At the second interim analysis, the DSMC performed an analysis of 28-day mortality and could have recommended stopping the trial to the steering committee at a 2-sided *P* value <.001. This Haybittle-Peto stopping boundary only trivially inflates the overall type I error, so a *P* value <.05 will be used to declare statistical significance in the final analysis [20].

After both interim analyses, the DSMC recommended continuation of enrollment as planned.

#### Intention-to-Treat Principle

We will analyze data from participants in the group to which they were allocated irrespective of protocol adherence. If ineligible participants were randomized, we will allow postrandomization exclusions only if they meet all of the following conditions: (1) the information about ineligibility was available at randomization; (2) participants did not receive the assigned intervention; (3) blinding was maintained; and (4) 2 members of the steering committee blinded to allocation agree that the participant was mistakenly randomized after review of information from medical records available at the time of randomization [21,22]. Patients who withdraw consent for their follow-up data to be used will also be excluded from the analyses.

#### Other Principles

The RCT will be analyzed using a frequentist approach. All statistical tests will be 2-sided, and the overall type 1 error for the primary outcome will be 5% at a significance level of .05. We will not report *P* values for secondary outcomes and analyses. All estimates of treatment effect will be reported with 95% CIs.

Categorical variables will be summarized with counts and percentages (based on the number of patients with data), and continuous variables will be reported as mean (SD) or median (IQR) as appropriate.

The main LOVIT manuscript will include analyses of the primary outcome and all secondary efficacy and safety outcomes, except for procalcitonin and C-reactive protein, as noted above. Unadjusted and adjusted analyses of the primary outcome and of 28-day mortality will be reported (see below); analyses of all other secondary outcomes will be unadjusted for baseline covariates.

Secondary outcome analyses will be performed regardless of the result for the primary outcome and will be considered exploratory.

Subgroup analyses will be performed regardless of the result for the primary outcome.

Analyses will be conducted using SAS 9.4 (SAS Institute Inc) and R 4.0.3 (R Foundation for Statistical Computing).

#### Trial Profile

**Figure 1:**
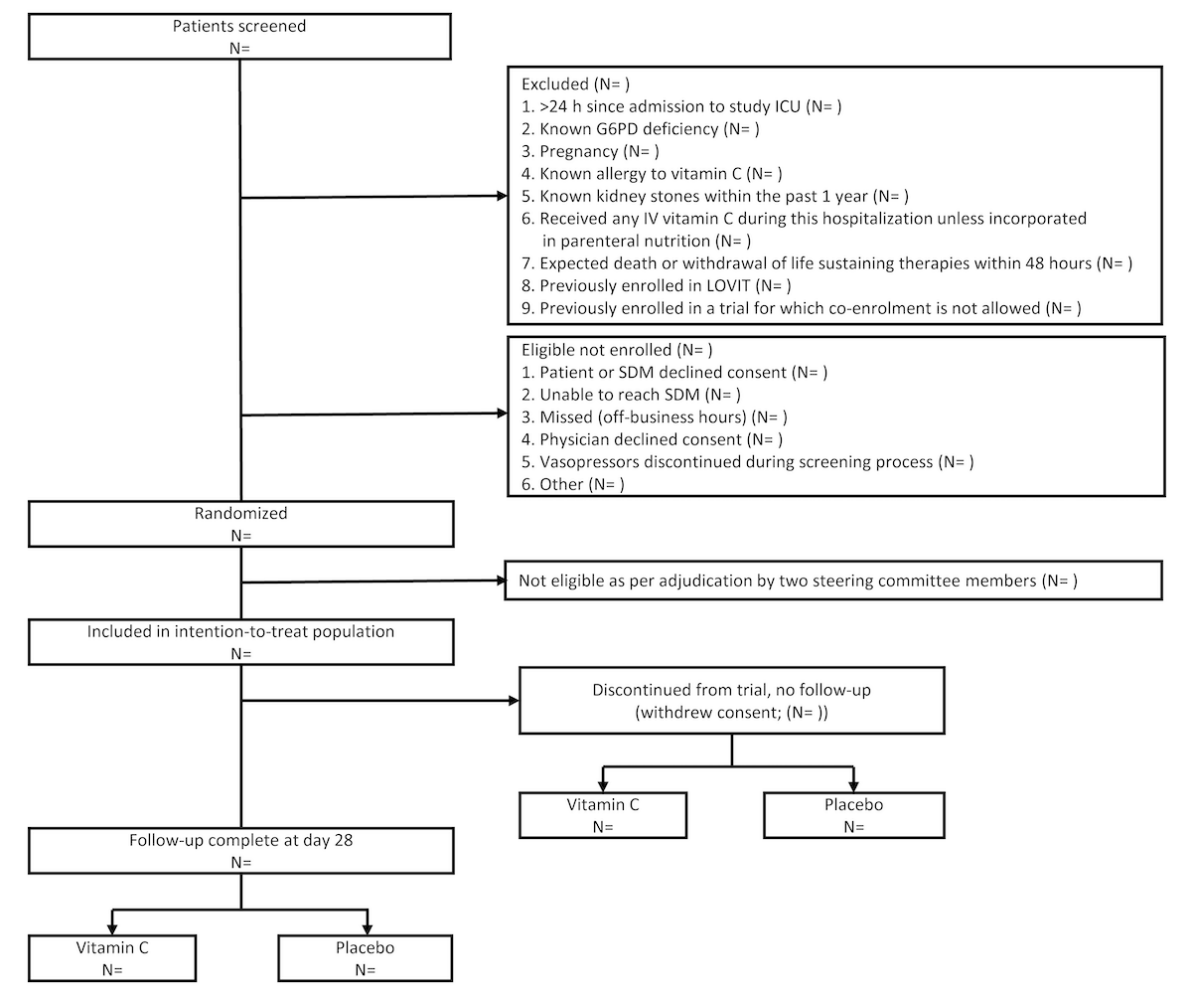
Flow of patients through the trial. G6PD: glucose-6-phosphate dehydrogenase; IV: intravenous; LOVIT: Lessening Organ Dysfunction with Vitamin C; SDM: substitute decision-maker.

The flow of patients through the trial will be shown in a CONSORT (Consolidated Standards of Reporting Trials) figure (Figure 1) [23]. The figure will show the number of patients who fulfilled eligibility criteria, the number randomized, and the number analyzed for the primary outcome. Reasons for eligible patients not randomized and for exclusion after randomization will be given.

#### Baseline Characteristics

**Figure 2:**
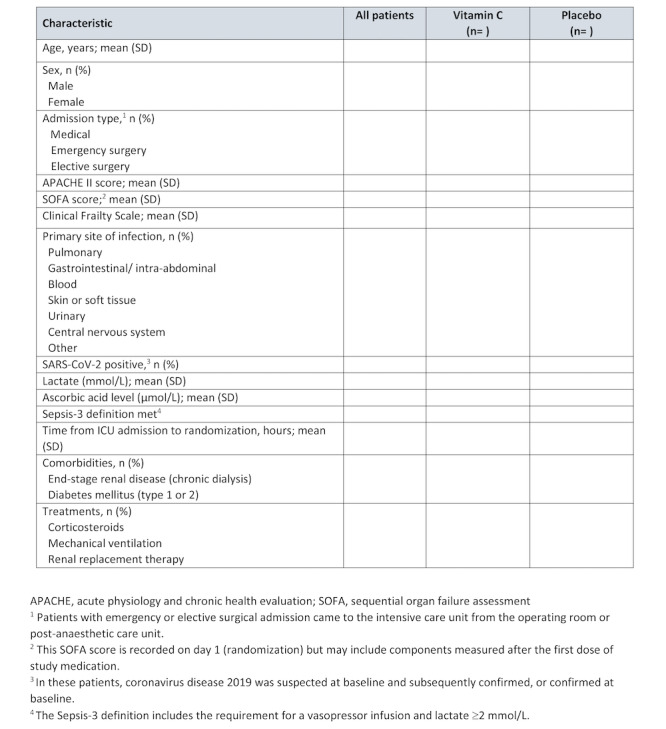
Table 1 in the main manuscript (baseline characteristics).

A table (Figure 2) will be used to display baseline characteristics for the entire trial population and according to the allocated group. These characteristics will include demographics, comorbidities, location of suspected infection, severity of illness, organ support (mechanical ventilation and renal replacement therapy), and laboratory data.

#### Adherence to the Protocol

Protocol adherence will be defined by the administration of at least 90% of scheduled doses of the study medication (vitamin C or placebo), until completion of the treatment protocol or ICU discharge, whichever comes first, and off-protocol administration of intravenous vitamin C.

#### Analysis of the Primary Outcome

For the principal analysis, we will report the number and percentage of patients who die or have persistent organ dysfunction at day 28. We will estimate the risk ratio and 95% CI in a generalized linear mixed model with binomial distribution and log link, with site as a random effect [24]. If this model does not converge, we will estimate the risk ratio using modified Poisson regression with small sample correction [25], and if that model also does not converge, we will estimate the odds ratio with logistic regression; both models will consider site as a random effect. We will use the same approach for the secondary analyses of the primary outcome and for analyses of binary secondary outcomes.

In secondary analyses of the primary outcome, we will adjust for prespecified baseline characteristics (age, sex, Acute Physiology and Chronic Health Evaluation [APACHE] II score [26], baseline receipt of corticosteroids, and time from ICU admission to randomization). Continuous adjustment variables will be modeled using restricted cubic splines with 4 knots to account for nonlinear relationships with the log risk of the primary outcome. If more than 5% of the intention-to-treat population is excluded from this adjusted analysis because of missing baseline characteristics, we will impute missing data using multiple imputation with fully conditional specification to obtain 10 imputed data sets. The adjusted analysis will be performed on the imputed data sets, and the results will be pooled using Rubin rules so that both within- and between-imputation variances are counted. We will assume the APACHE II score is missing only if all its components are missing; otherwise, we will assume that a missing component has a normal value and calculate the APACHE II score accordingly.

For patients with missing data on the primary outcome or on the secondary outcome of 28-day mortality (eg, due to loss to follow-up), the principal and adjusted analyses will only include data on patients with outcome data. We will conduct a best case-worst case unadjusted sensitivity analysis, assuming first that all patients with missing data who received vitamin C did not have the outcome, whereas those in the placebo group did, and assuming second that the opposite states apply. If these analyses give discrepant results, namely statistically significant in one case but not the other or both statistically significant but in opposite directions, then we will use multiple imputation with fully conditional specification to explore the impact of missing data [27]. We acknowledge the limitations of this approach, given that outcome data may not be missing completely at random, and the importance of making all efforts to minimize the extent of missing outcome data.

#### Subgroup Analyses

We will evaluate the effect of vitamin C on the primary outcome in subgroups defined at baseline by age (<65 vs ≥65 years), sex (male vs female), frailty (Clinical Frailty Scale 1-4 vs ≥5 [28]), severity of illness (quartiles of predicted risk of death from the baseline APACHE II score), Sepsis-3 [1] definition of septic shock (vasopressor infusion required to maintain a mean arterial pressure of 65 mmHg and lactate ≥2 mmol/L vs vasopressor need alone), and baseline ascorbic acid level (as quartiles). We hypothesize that vitamin C is more beneficial in elderly patients, those with greater frailty and illness severity at baseline, those who meet strict criteria for septic shock, and those with lower baseline ascorbic acid levels. In addition to the 6 subgroups prespecified in our published protocol, we will assess for a subgroup effect based on COVID-19 status (positive result on polymerase chain reaction or a rapid antigen test at baseline vs negative), hypothesizing no evidence of a difference in treatment effect. We will report interaction terms from the generalized linear mixed model (as used in the principal analysis) with treatment group, subgroup, and their interaction, and display the results in a Forest plot. We will assess the credibility of any subgroup effect with interaction at *P*<.05, using a published tool [29].

#### Analyses of Secondary Outcomes

Unless noted, analyses will not be adjusted for baseline characteristics or for site.

Analyses of clinical secondary outcomes will proceed as presented below.

#### Mortality at Day 28

We will conduct a principal unadjusted analysis and secondary analysis adjusted for baseline characteristics and site according to the analysis plan for the primary outcome outlined above. We will also conduct a best case-worst case unadjusted sensitivity analysis to account for missing data, with multiple imputation for missing outcome data if these 2 sensitivity analyses differ (as for the primary outcome).

#### Six-Month Mortality

We will conduct a principal analysis using a Cox proportional hazards model, with site as a random effect. We have chosen a Cox model because we record the data of death for decedents and because differences in duration of survival are plausibly important over a 6-month time horizon. Patients who are lost to follow-up or who withdraw consent for follow-up will be censored at the last follow-up time (expected to be at hospital discharge).

#### Six-Month HRQoL

In survivors with complete follow-up, we will report the mean or median for each dimension of the scale and for the self-reported overall health status in each group. Differences in means or medians will be reported, as appropriate.

#### Persistent Organ Dysfunction–Free Days in the ICU (up to Day 28)

Analysis will be rank-based, with death assigned as −1 (modified from a previous report [7]). We will display an empirical cumulative distribution function for each group and report the median, along with a difference in medians.

#### SOFA Scores at Prespecified Time Points

Results by randomized group at each time point will be summarized descriptively and displayed in a boxplot. For scores during the first 7 days, we will use a linear mixed model to account for repeated measures, with a random intercept and time for each subject and a random effect for site. Because day 1 SOFA may not be a true baseline value, it will not be used to model SOFA on subsequent days. For patients who die before day 7, we will impute the worst (highest) value, and for patients discharged alive before day 7, we will impute the value based on data available for these patients. We will conduct a likelihood ratio test between the empty model and the one with time, group, and their interaction, and will conduct additional testing of the terms in the model only if that test is statistically significant. For SOFA scores beyond day 7, we will report differences in means or medians because of the expected large proportion of patients with missing data due to death or discharge from the ICU.

For each biomarker outcome, results by randomized group at each time point will be summarized descriptively and displayed in boxplots. We will use constrained longitudinal data analysis [30] to analyze biomarker results. At day 3 and day 7, groups will be compared using a linear mixed model and adjusting for day 1 biomarker levels, with a random intercept for site and unstructured within-patient covariance. Biomarker data will be transformed, if necessary, to satisfy model assumptions.

For safety outcomes, we will report the number and percentage of each prespecified safety outcome, and the number of unexpected serious adverse events and number of patients with an unexpected serious adverse event, in each treatment group. Differences will be reported as risk ratios.

#### Tables and Figures

**Figure 3:**
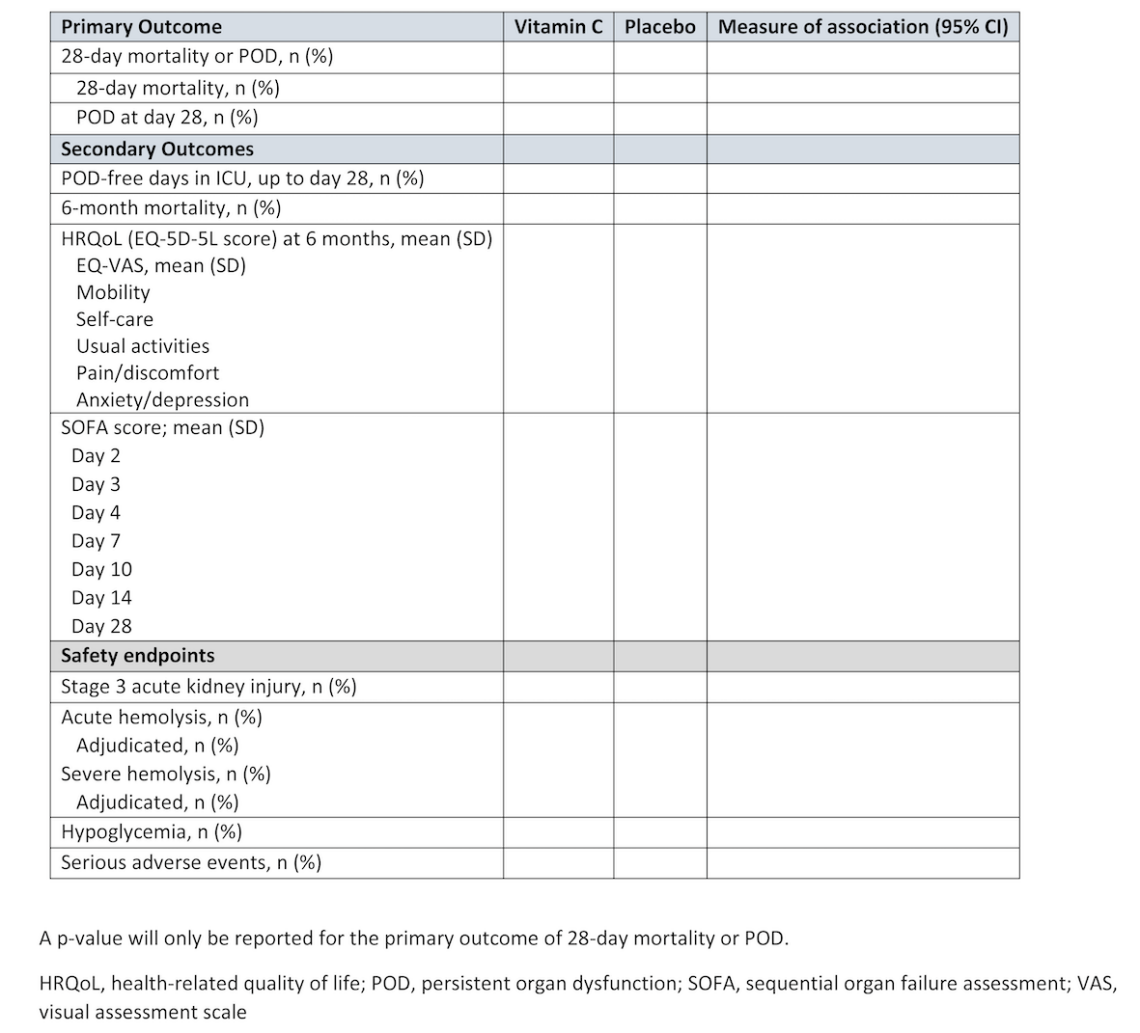
Table 2 in the main manuscript (primary and secondary outcomes).

Draft tables (Figures 2 and 3) and figures (Figure 1; Textbox 1) for the main manuscript are presented, and planned additional tables and figures are described in Multimedia Appendix 1.

**Additional figures planned for the main manuscript.**

- Sequential organ failure assessment scores over days 1-7 in the vitamin C and placebo groups (displayed as a boxplot)
- Subgroup analyses (displayed as a Forest plot)

### Funding, Registration, and Ethics Approval

The LOVIT trial is funded by a grant from the Lotte and John Hecht Memorial Foundation (grant 4318). The funder had no role in the design of the study, ongoing data collection, SAP or data interpretation, or writing of any associated manuscript.

The LOVIT trial was conducted with the support of the Canadian Critical Care Trials Group. The protocol has been approved by the Comité d’éthique de la recherche du Centre intégré universitaire de santé et de services sociaux de l’Estrie – Centre hospitalier universitaire de Sherbrooke (reference MP-31-2019-2945) and at each participating site. The LOVIT trial has been registered on ClinicalTrials.gov (NCT03680274; September 21, 2018).

### Document History

Version 1 of the statistical analysis protocol, dated January 19, 2022, was posted to this trial’s listing on ClinicalTrials.gov on January 20, 2022. This version contains corrections and clarifications, but no changes to the proposed analysis methods.

## Results

As of July 19, 2021, enrollment in the trial has been completed. Follow-up data at 6 months were available by January 24, 2022, with results to follow based on this SAP.

## Discussion

The LOVIT trial is a methodologically rigorous RCT of intravenous vitamin C monotherapy in critically ill patients with sepsis. This SAP, drafted before data collection was complete for the last patient enrolled in the trial and prior to database lock and unblinding of the study team, will guide the analysis of data from this trial.

## Appendix

Multimedia Appendix 1 Planned supplementary tables and figures.

## Abbreviations

APACHE: Acute Physiology and Chronic Health Evaluation
DSMC: Data and Safety Monitoring Committee
HRQoL: health-related quality of life
ICU: intensive care unit
LOVIT: Lessening Organ Dysfunction with Vitamin C
RCT: randomized clinical trial
SAP: statistical analysis plan
SOFA: sequential organ failure assessment

## References

[1] Singer M, Deutschman CS, Seymour CW, Shankar-Hari M, Annane D, Bauer M, Bellomo R, Bernard GR, Chiche J, Coopersmith CM, Hotchkiss RS, Levy MM, Marshall JC, Martin GS, Opal SM, Rubenfeld GD, van der Poll T, Vincent J, Angus DC (2016). The Third International Consensus Definitions for Sepsis and Septic Shock (Sepsis-3). JAMA.

[2] Marshall JC (2014). Why have clinical trials in sepsis failed?. Trends Mol Med.

[3] Langlois PL, Manzanares W, Adhikari NKJ, Lamontagne F, Stoppe C, Hill A, Heyland DK (2019). Vitamin C Administration to the Critically Ill: A Systematic Review and Meta-Analysis. JPEN J Parenter Enteral Nutr.

[4] Fujii T, Salanti G, Belletti A, Bellomo R, Carr A, Furukawa TA, Luethi N, Luo Y, Putzu A, Sartini C, Tsujimoto Y, Udy AA, Yanase F, Young PJ (2022). Effect of adjunctive vitamin C, glucocorticoids, and vitamin B1 on longer-term mortality in adults with sepsis or septic shock: a systematic review and a component network meta-analysis. Intensive Care Med.

[5] Masse M, Ménard J, Sprague S, Battista M, Cook DJ, Guyatt GH, Heyland DK, Kanji S, Pinto R, Day AG, Cohen D, Annane D, McGuinness S, Parke R, Carr A, Arabi Y, Vijayaraghavan BKT, D'Aragon F, Maslove D, Hunt M, Rochwerg B, Millen T, Chassé M, Lebrasseur M, Archambault P, Deblois E, Drouin C, Lellouche F, Lizotte P, Watpool I, Porteous R, Clarke F, Marinoff N, Belley-Côté É, Bolduc B, Walker S, Iazzetta J, Adhikari NKJ, Lamontagne F, Carbonneau, Canadian Critical Care Trials Group (2020). Lessening Organ dysfunction with VITamin C (LOVIT): protocol for a randomized controlled trial. Trials.

[6] Heyland DK, Muscedere J, Drover J, Jiang X, Day AG, Canadian Critical Care Trials Group (2011). Persistent organ dysfunction plus death: a novel, composite outcome measure for critical care trials. Crit Care.

[7] Goligher EC, Bradbury CA, McVerry BJ, Lawler PR, Berger JS, Gong MN, Carrier M, Reynolds HR, Kumar A, Turgeon AF, Kornblith LZ, Kahn SR, Marshall JC, Kim KS, Houston BL, Derde LPG, Cushman M, Tritschler T, Angus DC, Godoy LC, McQuilten Z, Kirwan BA, Farkouh ME, Brooks MM, Lewis RJ, Berry LR, Lorenzi E, Gordon AC, Ahuja T, Al-Beidh F, Annane D, Arabi YM, Aryal Di, Baumann Kreuziger L, Beane A, Bhimani Z, Bihari S, Billett HH, Bond L, Bonten M, Brunkhorst F, Buxton M, Buzgau A, Castellucci LA, Chekuri S, Chen JT, Cheng AC, Chkhikvadze T, Coiffard B, Contreras A, Costantini TW, de Brouwer S, Detry MA, Duggal A, Džavík V, Effron MB, Eng HF, Escobedo J, Estcourt LJ, Everett BM, Fergusson DA, Fitzgerald M, Fowler RA, Froess JD, Fu Z, Galanaud JP, Galen BT, Gandotra S, Girard TD, Goodman AL, Goossens H, Green C, Greenstein YY, Gross PL, Haniffa R, Hegde SM, Hendrickson CM, Higgins AM, Hindenburg AA, Hope AA, Horowitz JM, Horvat CM, Huang DT, Hudock K, Hunt BJ, Husain M, Hyzy RC, Jacobson JR, Jayakumar D, Keller NM, Khan A, Kim Y, Kindzelski A, King AJ, Knudson M, Kornblith A, Kutcher M, Laffan M, Lamontagne F, Le Gal G, Leeper C, Leifer E, Lim G, Gallego Lima F, Linstrum K, Litton E, Lopez-Sendon J, Lother S, Marten N, Saud Marinez A, Martinez M, Mateos Garcia E, Mavromichalis S, McAuley D, McDonald E, McGlothlin A, McGuinness S, Middeldorp S, Montgomery S, Mouncey P, Murthy S, Nair G, Nair R, Nichol A, Nicolau J, Nunez-Garcia B, Park J, Park P, Parke R, Parker J, Parnia S, Paul J, Pompilio M, Quigley J, Rosenson R, Rost N, Rowan K, Santos F, Santos M, Santos M, Satterwhite L, Saunders C, Schreiber J, Schutgens R, Seymour C, Siegal D, Silva D, Singhal A, Slutsky A, Solvason D, Stanworth S, Turner A, van Bentum-Puijk W, van de Veerdonk F, van Diepen S, Vazquez-Grande G, Wahid L, Wareham V, Widmer R, Wilson J, Yuriditsky E, Zhong Y, Berry S, McArthur C, Neal M, Hochman J, Webb S, Zarychanski R, REMAP-CAP Investigators, ACTIV-4a Investigators, ATTACC Investigators (2021). Therapeutic Anticoagulation with Heparin in Critically Ill Patients with Covid-19. N Engl J Med.

[8] Herdman M, Gudex C, Lloyd A, Janssen M, Kind P, Parkin D, Bonsel G, Badia X (2011). Development and preliminary testing of the new five-level version of EQ-5D (EQ-5D-5L). Qual Life Res.

[9] Gu W, Zhang Z, Bakker J (2015). Early lactate clearance-guided therapy in patients with sepsis: a meta-analysis with trial sequential analysis of randomized controlled trials. Intensive Care Med.

[10] Vincent JL, Moreno R, Takala J, Willatts S, De Mendonça A, Bruining H, Reinhart CK, Suter PM, Thijs LG (1996). The SOFA (Sepsis-related Organ Failure Assessment) score to describe organ dysfunction/failure. On behalf of the Working Group on Sepsis-Related Problems of the European Society of Intensive Care Medicine. Intensive Care Med.

[11] Fowler AA, Syed AA, Knowlson S, Sculthorpe R, Farthing D, DeWilde C, Farthing CA, Larus TL, Martin E, Brophy DF, Gupta S, Fisher BJ, Natarajan R, Medical Respiratory Intensive Care Unit Nursing (2014). Phase I safety trial of intravenous ascorbic acid in patients with severe sepsis. J Transl Med.

[12] Yeo TW, Lampah DA, Gitawati R, Tjitra E, Kenangalem E, Piera K, Price RN, Duffull SB, Celermajer DS, Anstey NM (2008). Angiopoietin-2 is associated with decreased endothelial nitric oxide and poor clinical outcome in severe falciparum malaria. Proc Natl Acad Sci U S A.

[13] Kidney Disease: Improving Global Outcomes (KDIGO) Acute Kidney Injury Work Group (2012). KDIGO Clinical Practice Guideline for Acute Kidney Injury. Kidney Int Suppl.

[14] Kahn SA, Lentz CW (2015). Fictitious hyperglycemia: point-of-care glucose measurement is inaccurate during high-dose vitamin C infusion for burn shock resuscitation. J Burn Care Res.

[15] Cook D, Lauzier F, Rocha MG, Sayles MJ, Finfer S (2008). Serious adverse events in academic critical care research. CMAJ.

[16] Marik PE, Khangoora V, Rivera R, Hooper MH, Catravas J (2017). Hydrocortisone, Vitamin C, and Thiamine for the Treatment of Severe Sepsis and Septic Shock: A Retrospective Before-After Study. Chest.

[17] Zabet MH, Mohammadi M, Ramezani M, Khalili H (2016). Effect of high-dose Ascorbic acid on vasopressor's requirement in septic shock. J Res Pharm Pract.

[18] Heyland D, Muscedere J, Wischmeyer PE, Cook D, Jones G, Albert M, Elke G, Berger MM, Day AG, Canadian Critical Care Trials Group (2013). A randomized trial of glutamine and antioxidants in critically ill patients. N Engl J Med.

[19] Orkin AM, Gill PJ, Ghersi D, Campbell L, Sugarman J, Emsley R, Steg PG, Weijer C, Simes J, Rombey T, Williams HC, Wittes J, Moher D, Richards DP, Kasamon Y, Getz K, Hopewell S, Dickersin K, Wu T, Ayala AP, Schulz KF, Calleja S, Boutron I, Ross JS, Golub RM, Khan KM, Mulrow C, Siegfried N, Heber J, Lee N, Kearney PR, Wanyenze RK, Hróbjartsson A, Williams R, Bhandari N, Jüni P, Chan A, CONSERVE Group (2021). Guidelines for Reporting Trial Protocols and Completed Trials Modified Due to the COVID-19 Pandemic and Other Extenuating Circumstances: The CONSERVE 2021 Statement. JAMA.

[20] Schulz KF, Grimes DA (2005). Multiplicity in randomised trials II: subgroup and interim analyses. The Lancet.

[21] Fergusson D, Aaron SD, Guyatt G, Hébert P (2002). Post-randomisation exclusions: the intention to treat principle and excluding patients from analysis. BMJ.

[22] Akl EA, Briel M, You JJ, Sun X, Johnston BC, Busse JW, Mulla S, Lamontagne F, Bassler D, Vera C, Alshurafa M, Katsios CM, Zhou Q, Cukierman-Yaffe T, Gangji A, Mills EJ, Walter SD, Cook DJ, Schünemann HJ, Altman DG, Guyatt GH (2012). Potential impact on estimated treatment effects of information lost to follow-up in randomised controlled trials (LOST-IT): systematic review. BMJ.

[23] Schulz KF, Altman DG, Moher D, CONSORT Group (2010). CONSORT 2010 statement: updated guidelines for reporting parallel group randomised trials. BMJ.

[24] Kahan BC (2014). Accounting for centre-effects in multicentre trials with a binary outcome - when, why, and how?. BMC Med Res Methodol.

[25] Yelland LN, Salter AB, Ryan P (2011). Performance of the modified Poisson regression approach for estimating relative risks from clustered prospective data. Am J Epidemiol.

[26] Knaus WA, Draper EA, Wagner DP, Zimmerman JE (1985). APACHE II: a severity of disease classification system. Crit Care Med.

[27] van Buuren S (2007). Multiple imputation of discrete and continuous data by fully conditional specification. Stat Methods Med Res.

[28] Rockwood K, Song X, MacKnight C, Bergman H, Hogan DB, McDowell I, Mitnitski A (2005). A global clinical measure of fitness and frailty in elderly people. CMAJ.

[29] Schandelmaier S, Briel M, Varadhan R, Schmid CH, Devasenapathy N, Hayward RA, Gagnier J, Borenstein M, van der Heijden GJMG, Dahabreh IJ, Sun X, Sauerbrei W, Walsh M, Ioannidis JPA, Thabane L, Guyatt GH (2020). Development of the Instrument to assess the Credibility of Effect Modification Analyses (ICEMAN) in randomized controlled trials and meta-analyses. CMAJ.

[30] Coffman CJ, Edelman D, Woolson RF (2016). To condition or not condition? Analysing 'change' in longitudinal randomised controlled trials. BMJ Open.

