# Supplementary material for: Lessening Organ Dysfunction With Vitamin C (LOVIT) Trial: Statistical Analysis Plan
Source: JMIR Res Protoc. 2022 May 20;11(5):e36261. doi: 10.2196/36261 (PMC9166642; doi:10.2196/36261)
Supplement: Multimedia Appendix 1 [file resprot_v11i5e36261_app1.pdf]

Lessening Organ dysfunction with VITamin C (LOVIT) Trial: statistical analysis plan
Supplement: planned additional figure and tables

Neill KJ Adhikari*
Ruxandra Pinto
Andrew Day
Marie-Hélène Masse
Julie Ménard
Sheila Sprague
Djillali Annane
Yaseen Arabi
Marie-Claude Battista
Dian Cohen
Deborah Cook
Gordon Guyatt
Daren Heyland
Salmaan Kanji
Shay McGuinness
Rachael Parke
Bharath Kumar Tirupakuzhi Vijayaraghavan
Emmanuel Charbonney
Michaël Chassé
Lorenzo del Sorbo
Demetrios Kutsogiannis
François Lauzier
Rémi LeBlanc
David Maslove
Sangeeta Mehta
Armand Mekontso Dessap
Tina Mele
Bram Rochwerg
Oleksa Rewa
Jason Shahin
Pawel Twardowski
Paul Young
François Lamontagne*

*contributed equally and corresponding authors; neill.adhikari@utoronto.ca and francois.lamontagne@usherbrooke.ca

Document history
Version 1: 19 January 2022

JMIR Res Protoc 2022;11(5):e36261

Planned supplementary figure

Figure S1 Boxplot of SOFA scores over after day 7

Table S1 Baseline characteristics

Characteristic	All patients	Vitamin C (n=)	Placebo (n=)
Age, years; mean (SD)			
Sex; n (%)<br>Male<br>Female			
Height (cm); mean (SD)			
Weight (kg);[1] mean (SD)			
Body mass index (kg/m^2); mean (SD)			
Admission type, n (%)<br>Medical<br>Emergency surgery<br>Elective surgery			
APACHE II score; mean (SD)			
SOFA score; mean (SD)[2]			
Clinical Frailty Scale; mean (SD)			
Primary site of infection; n (%)<br>Pulmonary<br>Gastrointestinal/ intra-abdominal<br>Blood<br>Skin or soft tissue<br>Urinary<br>Central nervous system<br>Other			
SARS-CoV-2 positive;[3] n (%)			
Mean arterial pressure, mmHg; mean (SD)			
Lactate (mmol/L); mean (SD)			
Ascorbic acid level (µmol/L); mean (SD)			
Sepsis-3 definition met; n (%)[4]			
Time from ICU admission to randomization, hours; mean (SD)			
Time from hospital admission to randomization, hours; mean (SD)			
Transferred from ICU of another hospital, n (%)<br>Time in other hospital ICU before admission to study ICU; mean (SD)			

**Characteristic**	**All patients**	**Vitamin C (n=)**	**Placebo (n=)**
Comorbidities,[5] n (%)			
Cardiac			
Supraventricular arrhythmia			
Angina or previous MI, CABG or PCI			
CHF class 1-3			
CHF class 4			
Ventricular arrhythmia			
Left ventricular ejection fraction (%), mean (SD)			
Vascular			
Known hypertension			
Cerebrovascular disease (TIA or stroke)			
Peripheral vascular disease or claudication			
Endocrine			
Diabetes (type 1 or 2)			
Renal			
Receiving chronic dialysis			
Baseline creatinine			
Gastrointestinal			
Moderate-to-severe liver disease			
Chronic lung disease			
Immunosuppression			
Neurologic			
Cognitive impairment/dementia			
Treatments, n (%)			
Corticosteroids,[6] n (%)			
Dexamethasone			
Prednisone			
Methylprednisolone			
Hydrocortisone			
Other			
Mechanical ventilation			
Renal replacement therapy			
Vasopressors (n, %) and dose (mean [SD])			
Norepinephrine (μg/kg/min)			
Phenylephrine (μg/min)			
Epinephrine (μg/kg/min)			
Vasopressin (units/hr)			
Dopamine (μg/kg/min)			
Norepinephrine equivalents[7] (μg/kg/min)			
Metaraminol (mg/hr)			

APACHE, acute physiology and chronic health evaluation; CABG, coronary artery bypass grafting; CHF, congestive heart failure; ICU, intensive care unit; MI, myocardial infarction; PCI, percutaneous coronary intervention; SOFA, sequential organ failure assessment; TIA, transient ischemic attack

[1] Weight was measured when available and otherwise taken from the patient or family, or estimated.

[2] This SOFA score is recorded on day 1 (randomization) but may include components measured after the first dose of study medication.

[3] In these patients, coronavirus disease 2019 was suspected at baseline and subsequently tested and confirmed, or confirmed at baseline.

[4] The Sepsis-3 definition includes the requirement for a vasopressor infusion and lactate ≥2 mmol/L.

[5] Baseline creatinine refers to closest outpatient creatinine in the last 12 months, or lowest inpatient creatinine from the current hospitalization if no outpatient value available. Moderate-to-severe liver disease refers to Child's B or C cirrhosis and documented portal hypertension; episodes of past upper gastrointestinal bleeding attributed to portal hypertension; or prior episodes of hepatic failure/encephalopathy/coma. Chronic lung disease refers to chronic restrictive, obstructive, or vascular disease resulting in severe exercise restriction, i.e., unable to climb stairs or perform household duties; or documented chronic hypoxia, hypercapnia, secondary polycythemia, severe pulmonary hypertension (>40 mmHg), or respiratory dependency. Immunosuppression refers to malignancy requiring chemotherapy treatment in last 3 months; or neutropenia (absolute neutrophil count <0.5 x 10^9/L); or receiving chronic immunosuppressive medications (azathioprime, cyclosporine, cyclophosphamde, tacrolimus, methotrexate, mycophenolate, anti-TNF agents, interleukin-2 agents) or transplantation (including stem cell) at any time; or HIV positive.

[6] Systemic (oral or IV) corticosteroids in the intensive care unit before randomization.

[7] Calculated according to the method in NEJM 2017; 377: 419–430.

Table S2 Protocol deviations

Type of deviation	All patients (n=)	Vitamin C (n=)	Placebo (n=)
	No. of deviations; No. (%) of patients with deviation		
Adherence			
Open-label administration of vitamin C			
Administration of ≥90% of scheduled doses of study medication			
Deviations			
First dose of study medication given >4 hrs after randomization			
≥1 dose of study medication missed			
Glucose monitoring to adjust insulin deviated from protocol			

Table S3 Cointerventions during the intensive care unit stay, including the day of randomization

Cointervention	All patients (n=)	Vitamin C (n=)	Placebo (n=)
Corticosteroids			
N (%)			
Days; mean (SD)			
Antimicrobials			
N (%)			
Days; mean (SD)			
Thiamine			
N (%)			
Days; mean (SD)			
Any sedation/analgesia infusion			
N (%)			
Days; mean (SD)			
Benzodiazepine infusion			
N (%)			
Days; mean (SD)			
Opioid infusion			
N (%)			
Days; mean (SD)			
Propofol infusion			
N (%)			
Days; mean (SD)			
Dexmedetomidine infusion			
N (%)			
Days; mean (SD)			
Any enteral or parenteral nutrition			
N (%)			
Days; mean (SD)			
Enteral nutrition			
N (%)			
Days; mean (SD)			
Parenteral nutrition			
N (%)			
Days; mean (SD)			
Oral intake			
N (%)			
Days; mean (SD)			
Insulin			
N (%)			
Days; mean (SD)			

Any blood product; n (%)<br>Red blood cell; n (%)<br>Frozen plasma, platelets, or cryoprecipitate; n (%)<br>Albumin; n (%)			

Data were collected for the first 28 days, up to and including the day of discharge from the ICU. For each co-intervention, N refers to the number of patients that ever received it.

Table S4 Fluid balance during the first 7 days in the intensive care unit stay, including the day of randomization

	Day 1		Day 2		Day 3		Day 4		Day 5		Day 6		Day 7	
	Vit C	Placebo	Vit C	Placebo	Vit C	Placebo	Vit C	Placebo	Vit C	Placebo	Vit C	Placebo	Vit C	Placebo
Urine output, mL; mean (SD); n														
Fluid balance, mL; mean (SD); n														

Vit, vitamin

The mean hours of data on day 1, which included randomization to 23h59 on the same calendar day, was xx (SD xx).

Table S5 Life-sustaining therapies in the intensive care unit and additional outcomes

	Total<br>(n=)	Vitamin C<br>(n=)	Placebo<br>(n =)
Life-sustaining therapy[1]			
Vasopressor infusion<br>N (%)<br>Days in survivors; mean [SD]; n<br>Days in non-survivors; mean [SD]; n			
Invasive mechanical ventilation<br>N (%)<br>Days in survivors; mean [SD]; n<br>Days in non-survivors; mean [SD]; n			
Non-invasive mechanical ventilation<br>N (%)<br>Days in survivors; mean [SD]; n<br>Days in non-survivors; mean [SD]; n			
Renal replacement therapy<br>N (%)<br>Days in survivors; mean [SD]; n<br>Days in non-survivors; mean [SD]; n			
Extracorporeal life support; n (%)			
Additional outcome			
Length of ICU stay, days (mean, SD; n)<br>All patients<br>ICU survivors<br>ICU non-survivors			
Readmission to the ICU on or before 28 days, n (%)			
Length of hospital stay,[2] days (mean, SD; n)<br>All patients<br>Hospital survivors<br>Hospital non-survivors			

ICU, intensive care unit.

[1] Data were collected for the first 28 days, up to and including the day of discharge from the ICU. For each life sustaining therapy, N refers to the number of patients that ever received it.
[2] Hospital stay is recorded for index stay in the study hospital only.

Table S6 Analyses of the primary outcome and of 28-day mortality

**Model**	**Risk Ratio (95% confidence interval)**
Principal analysis of primary outcome<br>(GLMM, binomial distribution, random effect for site)	
Secondary analysis	
Adjusted for baseline characteristics	
Unadjusted, best case scenario<br>Unadjusted, worst case scenario	
Principal analysis of 28-day mortality<br>(GLMM, binomial distribution, random effect for site)	
Secondary analysis	
Adjusted for baseline characteristics	

GLMM, generalised linear mixed model

Best case-worst case unadjusted sensitivity analysis assumes first that all patients with missing data who received vitamin C did not have the outcome, whereas those in the placebo group did (best case), and assuming second that the opposite states apply (worst case)

Table S7 Credibility of subgroup assessments using ICEMAN tool

Subgroup	A priori	Prior evidence	Interaction test	Small number of effect modifiers	Cutpoint for continuous variable	Other	Overall
Age (<65 vs. ≥65 yr)							
Sex (male vs female)							
Frailty (Clinical Frailty Scale 1-4 vs. ≥5)							
Severity of illness (quartiles of predicted risk of death)							
Sepsis-3 criteria (vasopressor and and lactate ≥2 mmol/L, vs vasopressor alone)							
Baseline vitamin C level (quartile)[1]							
COVID-19 at baseline (present or not)							

The ICEMAN tool (Instrument to assess the Credibility of Effect Modification Analyses) is available at CMAJ 2020; 192: E901-6.

The questions are as follows:
1: Was the direction of the effect modification correctly hypothesized a priori?
2: Was the effect modification supported by prior evidence?
3: Does a test for interaction suggest that chance is an unlikely explanation of the apparent effect modification?
4: Did the authors test only a small number of effect modifiers or consider the number in their statistical analysis?
5: If the effect modifier is a continuous variable, were arbitrary cut points avoided?
6 Optional: Are there any additional considerations that may increase or decrease credibility?
7: How would you rate the overall credibility of the proposed effect modification?

[1] The p-value for the interaction between baseline vitamin C and treatment group was 0.xx; results for quartiles of vitamin C level are shown in Figure 3.

Table S8 Biomarker results

Biomarker	Day 1		Day 3		Day 7	
	Vitamin C (n=)	Placebo (n=)	Vitamin C (n=)	Placebo (n=)	Vitamin C (n=)	Placebo (n=)
Global tissue dysoxia<br>Lactate (mmol/L)						
Inflammation<br>IL-1ß (pg/ml)<br>TNF-α (pg/ml)						
Endothelial injury<br>TM (ng/ml)<br>ANG-2 (pg/ml)						

Day 1 samples were collected before administration of study medication. Due to delays in obtaining assays to measure procalcitonin and C-reactive protein, analyses of these planned biomarkers may be delayed and reported after the primary publication.

ANG-2, Angiopoietin-2; IL-1ß= Interleukin-1 beta; TM= Thrombomodulin; TNF-α= Tumor necrosis factor-alpha.
